# Effect of human chorionic gonadotropin injection before frozen-thawed embryo transfer: A retrospective cohort study

**DOI:** 10.1097/MD.0000000000035658

**Published:** 2023-12-01

**Authors:** Xin Xin, Li Dong, Lu Guan, Yixuan Wang, Jiaxi Li, Fang Lian

**Affiliations:** a Shandong University of Traditional Chinese Medicine, Jinan City, Shandong Province, China; b Affiliated Hospital of Shandong University of Traditional Chinese Medicine, Jinan City, Shandong Province, China.

**Keywords:** frozen-thawed embryo transfer, hormone replacement cycle, human chorionic gonadotropin injection

## Abstract

The aim of this study is to investigate the effect of human chorionic gonadotropin (hCG) in hormone replacement regimen for frozen-thawed embryos. We performed a retrospective cohort study and included patients who underwent frozen embryo transfer (FET) between January 1, 2020 and May 31, 2022. According to the protocols for the FET cycle, the patients were divided into control (n = 238) and hCG groups (n = 216). The clinical pregnancy rate, live birth rate, early abortion rate, late abortion rate, and ectopic pregnancy rate were compared between the 2 groups. There was a significant difference in clinical pregnancy rate between the hCG and control groups (55.1% vs 45.8%, *P* = .048). The ectopic pregnancy rate decreased (5.0% vs 6.4%, *P* = .654), while the live birth rate increased (36.1% vs 29.0%, *P* = .105) in the hCG group. However, these differences were not statistically significant. The administration of hCG injection in HRT-FET cycles alone was also found to be associated with clinical pregnancy by logistic regressive analysis. HCG injection in the hormone replacement regimen for FET increased the clinical pregnancy rate.

## 1. Introduction

Assisted reproductive technology is increasingly used to solve the problems related to human reproduction. In vitro fertilization/intracytoplasmic sperm injection and embryo transfer have become a mainstay for treatment for infertility.^[1]^ With mature technology available to process frozen and thawed embryos, frozen-thawed embryo transfer and fresh embryo transfer have gradually become the conventional treatment approach.

Frozen-thawed embryo transfer refers to the transfer of thawed embryos that were frozen for any indication. Some patients may have already undergone a fresh transfer that was previously successful and are returning to expand their family, or their previous transfer was unsuccessful. Freeze-all approach refers to the decision to freeze all embryos immediately following an egg retrieval.^[2]^ Frozen embryo transfer (FET) can be planned either in a “pure” natural cycle, modified natural cycle, stimulated or hormonal replacement therapy cycle. At present, the optimal means to prepare the endometrium for FET remain a matter of debate. Despite the ongoing controversy over the effectiveness and safety of FET and fresh embryo transfer, the development of frozen-thawed embryo transfer technology is still good news for patients not eligible for fresh embryo transfer. Importantly, it could increase the cumulative pregnancy rate of patients, optimize endometrial receptivity, allow time for preimplantation genetics testing, and facilitate fertility preservation.

Hormone replacement therapy is selected by most reproductive centers to promote endometrial proliferation during the FET cycle. Human chorionic gonadotropin (hCG) is believed to promote follicular maturation, support corpus luteum development, and prepare conditions for pregnancy. Considering the inhibitory effect of high estrogen level on follicular development, few reproductive physicians in the center give hCG injection before endometrial transformation during hormone replacement program. In spite of this, there are still a few doctors who give hCG injection to simulate the role of corpus luteum support during the endometrial transition period in order to fit the human natural pregnancy changes as much as possible. In order to verify the clinical value of the application of hCG in the process of replacing the endometrial transition of the cycle in the process of FET, it is urgent to carry out statistical analysis of the pregnancy rate of patients under different programs of the center and explore its potential significance, so as to optimize the selection of clinical programs.

In this retrospective study, patients underwent FET were selected, and the success rate of hormone replacement therapy frozen-thawed embryo transfer (HRT-FET) was observed. We sought to optimize the parameters of the hormone replacement cycle for subsequent FET.

## 2. Materials and methods

### 2.1. Subjects

This retrospective, cohort study was conducted at the Reproductive and Genetic Center of Provincial public tertiary comprehensive hospital, Shandong, China. This study was reviewed and approved by the Research ethics committee of the Affiliated Hospital of Shandong University of Traditional Chinese Medicine (ref approval no. SDUTCM20211003). Patients who underwent FET in our hospital from January 1, 2020 to May 31, 2022, were included in the study. All included patients provided written informed consent for the use of their data. All procedures were carried out in accordance with relevant guidelines and regulations and with the 1964 Helsinki declaration and its later amendments or comparable ethical standards.

Patients older than 45 years at the onset of the cycle, with basal follicle-stimulating hormone level > 12 U/L, and with uterine malformation, endometriosis, PCOS patients with severe insulin resistance and hyperandrogenemia, hydrosalpinx, recurrent spontaneous abortion, acute infection of the urogenital system and submucosal fibroid, endometrial polyps or intrauterine adhesions, either partner of couple has chromosomal abnormalities were all excluded from the study. We also removed patients who had more than one transfer and had successful FET pregnancy after fresh embryo transfer in the designated time period and those underwent preimplantation genetic testing for aneuploidy, while patients have removed submucosal fibroids or polyps (i.e., normal uterine cavity) were included in the study. Finally, data on 454 women were collected to observe the value of clinical pregnancy. The patients were divided into control (n = 238) and hCG groups (n = 216) based on hCG injection in the protocols. According to previous research results and formula calculations,^[3]^ at least 71 patients should be included. In this study, a total of 454 patients should have met the sample size requirements.

### 2.2. Endometrial preparation

Patients in the control and hCG groups both received hormone replacement regimens for FET. Oral estradiol valerate (Progynova, Delpharm Lille) at a dose of 4 mg/day was initiated on day 2 or 3 of the menstrual cycle for 3 to 4 days, according to the endometrial thickness and basal follicle-stimulating hormone, luteinizing hormone (LH), estradiol levels. Then, the dose was increased to 6 mg/day for 3 to 4 days. Finally, the dose of estrogen was maintained at 8 mg/day to promote endometrial proliferation. Endometrial thickness and endometrial pattern were monitored by vaginal B-ultrasound. During this cycle, the dosage and duration of estrogen were increased until the endometrial thickness reached an appropriate state for embryo transfer (generally at least 8 mm). Then the hCG group received an intramuscular injection of hCG (Zhuhai Lizhu company) 2000 U from 10:00 am to 12:00 am immediately, and received progesterone on the next day, while the control group only received progesterone administration for endometrial transformation on the next day until embryos or blastocysts transferation. Both groups received intramuscular injections of progesterone (progesterone injection, 20 mg b.i.d., Zhejiang Xianju Pharmaceutical Co., Ltd.), oral estradiol valerate 4 mg/day and oral hydroxyprogesterone 20 mg/day (Dapton, 10 mg b.i.d., Abbott, the Netherlands).

### 2.3. Embryo transfer and luteal phase support

High-quality embryos were defined according to the embryo morphology assessment by the Istanbul consensus workshop^[4]^ and blastocysts morphologic criteria.^[5]^ Proposed thawing time, embryo numbers (1 or 2) and embryo transfer time. It is believed that single embryo transfer can reduce the rate of multiple pregnancies at present. However, patients treated with hCG injection of our center in the past had cases of single transfer of 2 cleavage stage embryos or 1 blastocyst. Considering that our study intended to extensively observe the clinical value of using hCG injection on the day of endometrial transition in HRT-FET schemes, we included patients with a single transfer of ≤2 embryos depending on the embryo quality and patient age. Although it increased the risk of multiple pregnancies to some extent. At least one high-quality embryo was transferred. Three or 5 days after progesterone administration, synchronized warm embryos were transferred, the cleavage-stage embryos were transferred on the 4th day of endometrial transformation, while the blastocysts were transferred on the 6th day. The embryos or blastocysts were thawed on the day of transferation and transferred under abdominal ultrasound guidance.

Our center intended to choose a combination of estrogen and progesterone therapy for luteal support in patients undergoing HRT to facilitate embryo implantation and early pregnancy maintenance. After transferation, the same medication regimen (oral estradiol valerate + oral hydroxyprogesterone + progesterone injection) was continued to maintain luteal phase function. The blood hCG was measured 14 days after the frozen-thawed embryo transfer to determine biochemical pregnancy. If intrauterine clinical pregnancy was confirmed, estrogen support was continued until 7 weeks of gestation, and progestin supplementation was continued until 10 weeks of gestation.

### 2.4. Outcomes

Clinical pregnancy was determined when one or more gestational sac were found on vaginal ultrasound 4 weeks after transferation. Clinical pregnancy rate is defined as the number of pregnancy cycles divided by the total number of transplant cycles. The early abortion rate was defined as the percentage of miscarriages occurring earlier than 12 weeks divided by the total number of clinical pregnancy cycles. The late abortion was the proportion of miscarriages occurring between 12 and 28 weeks. The preterm birth was defined as the percentage of birth before 34 weeks in women with live birth. Live birth rate was defined as the number of deliveries of at least one living baby divided by the total number of transplant cycles. Multiple pregnancy rate was defined as the number of cycles of more than one embryo discovered under ultrasound divided by the total number of transfer cycles. The differences in endometrium thickness, clinical pregnancy rate, abortion rate, preterm birth rate, live birth rate and multiple pregnancy rate were compared. The clinical pregnancy rate was the primary outcome. Considering that some of the enrolled patients were in the recent embryo transfer cycle, which were only followed up to the stage of clinical pregnancy, the live birth rate and abortion rate were not the primary outcomes of the study.

### 2.5. Statistical analysis

Continuous variables were expressed as means and standard deviations; differences in variables were compared using Student *t* test. Categorical variables were described as frequencies and percentages, with the between-group difference tested using the chi-square test and Fisher exact test when the number of events was <5. Multivariate logistic regression analysis was utilized to examine the possible effects of the following known potential confounding factors on clinical pregnancy outcomes of FET cycle, including age at FET cycle, BMI, infertility years and type, endometrium thickness prior to FET, number of embryos transferred, total number of transfers, embryo stage at transfer. A *P* value < .05 was considered statistically significant.

## 3. Results

### 3.1. Patients

The baseline characteristics were similar in the control and hCG groups (Table 1). There were no differences between the 2 groups in age, body mass index (BMI), basal endocrine hormone levels and anti-Müllerian hormone, and fertility years, duration of stimulation, Gonadotropin dosage, number of oocytes obtained, besides, the number of transferrable embryos in ovulation induction cycle, rate of transferring of embryo or blastocyst (embryo stage transferred), number of embryo transferred and the total number of transfer process of patients in 2 groups were not different. The indications for assisted reproduction of the 2 groups were all due to tubal or male factors, and patients with failed fresh embryo transfer. There were no significant differences in baseline patient characteristics between the hCG and control groups (*P* > .05).

**Table 1:** Basal characteristics of participants.

	Control group	hCG group	*P* value
Number	238	216	
Age (yr)	34.1 ± 4.7	34.6 ± 4.5	.182
AMH (ng/mL)	3.7 ± 1.8	3.7 ± 1.6	.938
BMI (kg/m^2^)	24.1 ± 3.9	23.7 ± 4.1	.303
Infertility years (yr)	2.9 ± 1.3	2.8 ± 1.5	.660
Basal FSH (U/L)	6.5 ± 2.1	6.7 ± 2.0	.245
Basal LH (U/L)	5.1 ± 2.5	5.3 ± 3.0	.532
Basal E2 (pg/mL)	45.8 ± 24.8	48.4 ± 28.6	.306
Basal P (ng/mL)	0.6 ± 0.3	0.6 ± 0.3	.453
Primary or secondary infertility			.287
Primary infertility	46.6% (111/238)	41.7%(90/216)	
Secondary infertility	53.4% (127/238)	58.3%(126/216)	
Duration of stimulation	10.7 ± 1.9	10.6 ± 1.9	.851
Gn dosage	2275.7 ± 682.4	2278.9 ± 672.1	.959
Number of oocytes obtained	11.7 ± 3.2	11.8 ± 3.1	.883
Number of transferrable embryo	6.1 ± 2.0	6.2 ± 2.0	.859
Transfer of embryo or blastocyst			.256
Embryo	72.7% (173/238)	77.3% (167/216)	
Blastocyst	27.3%(65/238)	22.7% (49/216)	
Total number of transfers			.126
First FET transfer after fresh embryo transfer	73.5%(175/238)	79.6% (172/216)	
Two or more FET transfer after fresh embryo transfer	26.5%(63/238)	20.4% (44/216)	

AMH = anti-Müllerian Hormone, BMI = body mass index, E2 = estrogen, FET = frozen-thawed embryo transfer, FSH = follicle-stimulating Hormone, Gn = gonadotrophin, LH = luteinizing hormone, P = progesterone.

### 3.2. Outcomes of FET

At least one high-quality embryo was transferred in each FET cycle in enrolled patients. The number of high-quality embryos, rate of receiving elective single embryo transfer versus multiple embryo transfer of 2 groups, and the rate of patients underwent cleavage stage embryo transfer versus blastocyst transfer did not differ significantly between the 2 groups. However, better outcomes were observed in the hCG group. The endometrium in the hCG group was thicker than in the control group. The clinical pregnancy rate of the hCG group was significantly higher than that of the control group (55.1% vs 45.8%, *P* = .048) (Table 2). Moreover, a higher live birth rate (36.1% vs 29.0%, *P* = .105), lower ectopic pregnancy rate (5.0% vs 6.4%, *P* = .654) were found in the hCG group compared to the control group. However, these differences were not statistically significant (Table 2). In addition, there were no significant differences in preterm birth rate (4.2% vs 4.6%, *P* = .887), early abortion rate (5.0% vs 10.0%, *P* = .147), late abortion rate (5.0% vs 5.5%, *P* = .876) and multiple pregnancy rate (15.1% vs 16.5%, *P* = .774) between the 2 groups (Table 2). A multivariate logistic regression model was used to analyze the clinical factors of the patients. We found that hCG treatment, infertility years or infertility type was significantly associated with clinical pregnancy rate in patients that underwent FET. While adjusted by hCG administration, age, BMI, infertility years and type, the number of transfer and number of embryos transferred, the thickness of the endometrium and embryo stage, the administration of hCG shows no relationship with primary outcome, although infertility years was still proved to be associated with clinical pregnancy (Table 3).

**Table 2:** Outcomes of frozen embryo transfer.

	Control Group	hCG Group	*P* value
Endometrium thickness	0.9 ± 0.1	1.0 ± 0.1	***<.01*** *
Number of transferred embryos			0.612
Single embryo transfer	40.8% (97/238)	38.4% (133/216)	
Multiple embryo transfer	59.2% (141/238)	61.6% (13/216)	
Number of high-quality embryos transferred	0.6 ± 0.7	0.5 ± 0.6	.780
Clinical pregnancy rate	45.8% (109/238)	55.1% (119/216) (119/216)	***.048*** *
Live birth rate	29.0% (69/238)	36.1% (78/21 6)	.105
Early abortion rate	10.1% (11/109)	5.0% (6/119)	.147
Late abortion rate	5.5%(6/109)	5.0% (6/119)	.876
Preterm birth rate	4.6% (5/109)	4.2% (5/119)	.887
Ectopic pregnancy rate	6.4% (7/109)	5.0% (6/119)	.654
Multiple pregnancy rate	16.5% (18/109)	15.1% (18/119)	.774

* *P* < .05.

**Table 3:** Multivariable logistic regression analysis.

	Crude model			Adjusted model		
	OR	95% CI	*P* value	OR	95% CI	*P* value
Group	0.682	0.471–0.987	***.043*** *	1.363	0.825–2.252	.227
Age	0.975	0.936–1.015	.220	0.960	0.918–1.003	.070
Infertility years	1.256	1.092–1.444	***.001*** *	1.285	1.104–1.496	***.001*** *
Infertility type	1.541	1.062–2.237	***.023*** *	1.356	0.916–2.015	.130
BMI	0.980	0.935–1.027	.401	0.980	0.934–1.029	.419
Endometrium thickness	7.815	0.787–77.625	.079	5.537	0.245–125.390	.282
Number of embryo transfer	0.909	0.624–1.324	.617	1.192	0.706–2.010	.511
Embryo or blastocyte	0.780	0.509–1.196	.255	0.648	0.362–1.196	.170
Total number of transfers	0.735	0.475–1.137	.167	0.820	0.519–1.294	.394

BMI = body mass index, CI = confidence interval, OR = odds ratio.

* *P* < .05.

## 4. Discussion

Clinical research and experience have shown that certain conditions were required to achieve highly successful pregnancy rates with FET.^[6,7]^ The most basic documented requirements are high-quality embryos and a good endometrial environment. High-quality cleavage embryos or blastocysts are initially selected for cryopreservation in the fresh cycle; the endometrium must then be prepared for optimal receptivity in FET. Nonetheless, many patients that undergo FET fail to get pregnant even if the pregnancy conditions are strictly met. In addition to considering the development potential of the embryo itself, emphasis should be laid on the endometrium preparation to ensure it is in synchrony with the embryo.

Herein, we found that adding hCG on the day of endometrium transformation significantly improved FET clinical pregnancy rate after a failed fresh embryo transfer cycle attempt. The live birth rate of the hCG group was also increased; however, this difference was not statistically significant.

No dominant follicle was released during the hormone replacement cycle while exogenous hCG addition triggered the LH peak. Animal and clinical experiments confirmed that LH reached its peak levels during the implantation window, which was considered a major factor that influenced the endometrial type and state changes,^[8]^ and played a significant role in regulating endometrial receptivity during transferation. Even though hCG and LH have a homologous α-subunit, hCG has a longer half-life, a better ability to bind to hCG/LH receptor in the endometrium,^[9]^ and is also cheap and widely used during clinical practice. During immunohistochemical localization and function of LH/hCG receptors in the endometrium, we found that LH and hCG receptors were highly expressed in endometrial glandular and stromal cells; their expression in the secretory endometrium was significantly higher than in the proliferative endometrium. Adding hCG stimulated the LH peak, which was beneficial for embryo implantation. Consistently, Wang et al found that low LH in the hormone replacement cycle of freeze-thaw embryo transfer was not conducive to embryo implantation and pregnancy maintenance; however, intramuscular injection of hCG on the day of endometrium transformation could improve the embryo implantation rate and clinical pregnancy rate, despite low LH levels.^[10]^ Studies also found that pregnancy success could be related to corpus luteum function during the FET cycle.^[3]^ In this regard, the lack of luteal function during pregnancy could be associated with adverse maternal and neonatal outcomes. In the present study, hCG injection on the day of endometrium transformation increased the clinical pregnancy rate and live birth rate, related to the stimulated LH peak by exogenous hCG. In terms of luteal phase support after pregnancy, hCG has been found to promote the expression of progesterone receptor-related proteins by activating extracellular signal-regulated protein kinases 1 and 2 and hence played a significant role in maintaining a pregnancy.^[11]^

It is widely acknowledged that hCG has numerous functions. The first step of embryo implantation is the “intimate dialogue” between embryo and endometrium at the maternal-fetal interface, mediated by various cytokines and related hormones. hCG is one of the molecules in the initial molecular information array between the embryo and the decidua. It is thought that hCG regulates intimal and placental angiogenesis; nonetheless, its mechanism of action remains clear. hCG receptors have been documented in the endometrium, and it has been hypothesized that hCG could directly stimulate angiogenesis. Drug release experiments have demonstrated that hCG could induce the proliferation of human umbilical vein endothelial cells and basic fibroblast growth factor in vitro through the p44/42MAPK pathway. Moreover, hCG could reportedly combine with adipose tissue-derived factors with angiogenesis characteristics (such as adipokines) to promote cell proliferation,^[12]^ which was beneficial to the implantation process.

Human studies have confirmed that hCG could regulate endometrial cell annexin IV’s mRNA and protein expression and vascular endothelial growth factor, promoting angiogenesis during implantation.^[13]^ Interestingly, clinical studies have shown decreased reproductive health index in obese individuals as hormone imbalances, fat toxicity, and expression of fat regulatory factors in obese people affected their gonads, peripheral reproductive organs and embryos.^[14]^ Importantly, the studies exploring the interaction between hCG and adipokines on angiogenesis in vitro also confirmed that maternal obesity was not conducive to angiogenesis of the uterus and placenta, which explained the potential relationship between obesity and infertility from another perspective.

Animal experiments have confirmed that hCG played an important role in regulating maternal immune tolerance during embryo implantation. In this regard, in vivo and in vitro experiments showed that hCG injection could promote communication of maternal T cells at the fetal-maternal interface during pregnancy by increasing the expression of inflammatory chemokine CCL2, which then improved maternal immune tolerance and facilitated embryo implantation.^[15]^ Furthermore, Bielfeld et al found that intrauterine hCG infusion could increase the expression of proteins related to endocrine, hypoxia-inducible factor 1 and various chemokines, which significantly improved the clinical pregnancy rate of patients with repeated implantation failures.^[16]^ In addition to the regulation of the above inflammatory factors to improve endometrial immune tolerance, hCG could also promote synchronous development of human embryo and endometrium by inducing the production of early decidual markers and promoting the development of endometrial glands and stroma; this process may occur by the stimulation of subcutaneous and perivascular myofibroblast marker antibodies α-SMA/ACTA2 expression.^[17]^

hCG has been reported as an early embryonic signal secreted before embryo implantation and a key factor in regulating the invasion of placental trophoblast, immune tolerance during embryo implantation^[18]^ and angiogenesis at the embryo implantation site.^[13,19]^ Importantly an increasing body of evidence suggests that hCG could regulate the expression of signal molecules related to endometrial receptivity.^[17,20]^

The blastomere of the fertilized egg transmits information to the endometrium through paracrine secretion of hCG in the early cell stage. For implanted embryos, exogenous addition may make up the loss of information and obstacles in transmission caused by freezing conducive to the information exchange between mother and embryo.^[21]^ Interestingly, Jenkins et al found that frozen embryos may increase the risk of cell variation, such as T lymphocyte telomere shortening.^[22]^ After embryo transfer, implantation could not be carried out due to poor immune tolerance. Importantly, hCG has been reported to induce secretion of transforming growth factor-β and Interleukin (IL-2, IL-10) by stromal cells, which can interact with the surface receptors of Treg, Breg, and DC cells. As the key endocrine and immune regulatory factors to start and maintain fetal tolerance, they regulated the local immune microenvironment of the endometrium, induced immune tolerance between mother and embryo, inhibited severe inflammatory response and enhanced endometrial receptivity,^[23,24]^ which was better for embryo implantation and maintaining pregnancy.

At present, most studies that explored the effects of intrauterine hCG perfusion before transferation validated its value in improving pregnancy success rates of FET^[25]^; however, due to the risks of endometrial injury or infection, an intramuscular injection of hCG could be more convenient. Similarly, previous studies found that hCG injections significantly improved pregnancy rates in endometriosis-associated infertility. It was speculated that hCG might enhance embryo implantation by interacting with molecules regulating endometrial receptivity.^[26]^

In the present study, we found a significant increase in the pregnancy rate. However, the difference of live birth rate was not statistically significant, which may be related to the limit of designate time of our study, that some patients did not give birth at the end of study, which led to a relative increase in the live birth rate that was not statistically significant. Long-term observation and longer follow-up of these patients are needed in future studies to substantiate our findings. We acknowledge limitations of our study. Ethyology and some demographic and clinical characteristics such as BMI and age are affecting assisted reproductive technology outcomes.^[27,28]^ We have not analyzed the causes of infertility between study groups and we acknowledge this as one of the limitations of the study. Crued-model analysis found that hCG injection before HRT-FET affects clinical pregnancy outcomes, but considering other factors such as age, infertility years, infertility type, and BMI of clinical patients and so on, the correlation is significantly reduced, the reason why it do not emphasize the important value of hCG application mostly because that there is no consensus on the dose and time of hCG injection which could be potential confounding factors. Further studies are necessary to corroborate the clinical significance of adding hCG and explore the effect of different doses to optimize the procedure and improve the success rate of FET. Finally, the correlation between the number of infertility years and clinical pregnancy rate mentioned in our experimental results could be attributed to patients with long-term infertility tend to experience higher anxiety levels, which resulted in worse pregnancy outcomes.^[29]^ In addition, our study is only a retrospective study with a single center and a small sample. In the future, prospective studies with multiple centers and a large sample are still needed to deeply explore the clinical value of hCG administration in the endometrial changes in function and morphology before HRT-FET.

## Author contributions

**Conceptualization:** Xin Xin.

**Data curation:** Li Dong, Lu Guan, Yixuan Wang, Jiaxi Li.

**Formal analysis:** Li Dong, Lu Guan.

**Funding acquisition:** Fang Lian.

**Investigation:** Li Dong, Lu Guan, Yixuan Wang, Jiaxi Li.

**Methodology:** Jiaxi Li.

**Supervision:** Fang Lian.

**Writing – original draft:** Xin Xin.

**Writing – review & editing**: Fang Lian.
